# Testing Gene-Gene Interactions Based on a Neighborhood Perspective in Genome-wide Association Studies

**DOI:** 10.3389/fgene.2021.801261

**Published:** 2021-12-08

**Authors:** Yingjie Guo, Honghong Cheng, Zhian Yuan, Zhen Liang, Yang Wang, Debing Du

**Affiliations:** ^1^ School of Electronic and Communication Engineering, Shenzhen Polytechnic, Shenzhen, China; ^2^ Institute of Fundamental and Frontier Sciences, University of Electronic Science and Technology of China, Chengdu, China; ^3^ School of Information, Shanxi University of Finance and Economics, Taiyuan, China; ^4^ Research Institute of Big Data Science and Industry, Shanxi University, Taiyuan, China; ^5^ School of Life Science, Shanxi University, Taiyuan, China; ^6^ Beidahuang Industry Group General Hospital, Harbin, China

**Keywords:** genome-wide association studies, qualitative traits, gene-gene interactions, maximal neighborhood coefficient, gene-based testing

## Abstract

Unexplained genetic variation that causes complex diseases is often induced by gene-gene interactions (GGIs). Gene-based methods are one of the current statistical methodologies for discovering GGIs in case-control genome-wide association studies that are not only powerful statistically, but also interpretable biologically. However, most approaches include assumptions about the form of GGIs, which results in poor statistical performance. As a result, we propose gene-based testing based on the maximal neighborhood coefficient (MNC) called gene-based gene-gene interaction through a maximal neighborhood coefficient (GBMNC). MNC is a metric for capturing a wide range of relationships between two random vectors with arbitrary, but not necessarily equal, dimensions. We established a statistic that leverages the difference in MNC in case and in control samples as an indication of the existence of GGIs, based on the assumption that the joint distribution of two genes in cases and controls should not be substantially different if there is no interaction between them. We then used a permutation-based statistical test to evaluate this statistic and calculate a statistical *p*-value to represent the significance of the interaction. Experimental results using both simulation and real data showed that our approach outperformed earlier methods for detecting GGIs.

## 1 Introduction

Genome-wide association studies (GWAS) has been used to investigate the associations between genetic variants and complex disorders with great success. Researchers have discovered more than 71,000 unique single nucleotide polymorphisms (SNPs) associated to diseases throughout the last decade (Hindorff et al., 2009; Zhang et al., 2016; Zeng et al., 2017; Guo et al., 2018; Buniello et al., 2019; Loos, 2020; Li et al., 2021). Traditional GWAS, on the other hand, concentrated on the independent, additive, and cumulative effects of individual SNPs on specific diseases. The majority of associated SNPs are common genetic variants with small effects that only explain a portion of complex disease heritability. Many genes, environmental variables, and interactions play a crucial role in the underlying genetic architecture of complex diseases (Cordell, 2009; Moore et al., 2010; Jiang et al., 2018; Liu et al., 2018; Liu et al., 2019a; Zhang et al., 2019; Chen et al., 2020; Luo et al., 2020; Liu et al., 2021; Shao et al., 2021; Su et al., 2021; Wang et al., 2021). As a result, genetic interactions are thought to enlighten studies into “missing heritability” (Manolio et al., 2009; Fang et al., 2019; Young, 2019; Tang et al., 2020; Song et al., 2021) and give important knowledge for constructing topologies for complex disease-related pathway.

Genetic interaction was originally explored at the SNP level, named epistasis. Methods (Li et al., 2015a; Ritchie and Van Steen, 2018; Lyu et al., 2020) can be classified into three categories based on their search strategy: exhaustive methods, searching methods, and machine learning-based methods, such as statistics based on entropy (Dong et al., 2008) and odds-ratios (Emily, 2012); MDR (Ritchie et al., 2003), BEAM (Zhang and Liu, 2007), BOOST (Wan et al., 2010), Epi-GTBN (Guo et al., 2019), GenEpi (Chang et al., 2020), and some accelerate methods (Nobre et al., 2021). For example, a logistic regression analysis revealed a significant interaction between the genes ERAP1 (rs27524) and HLA-C (rs10484554) in psoriasis ( $p=6.95\times 10^{-6}$ ), indicating that ERAP1 SNP was effective only in individuals who had at least one copy of the HLA-C SNP risk allele (Képíró et al., 2021). The statistical weakness of high-order or pairwise tests, which come from enormous multiple testing corrections over all pairs of SNPs, is one of the general problems of these marker-based approaches. Instead, we explored the interaction of two genes in a single gene-based interaction detection by treating SNPs inside a gene as a group.

The effectiveness of gene-based methods in GWAS marginal association studies should be extended to the study of gene-gene interaction (GGIs) (Emily, 2018; Emily et al., 2020). This strategy offers a number of possible benefits. For starters, it often has substantially fewer genes than SNPs, which dramatically decreases the number of pairwise testing. To discover GGIs in pair of 20,000 genes, for example, $∼2\times 10^{8}$ tests are necessary. However, for three million SNPs in a marker-based interaction, more than $5\times 10^{12}$ tests are required. Second, gene-based methods are more powerful statistically because a gene carries more information than individual SNP and genes interact in a variety of ways (Liu et al., 2010; Li et al., 2011; Jiang et al., 2017; Su et al., 2019; Hu et al., 2020; Hu et al., 2021a; Hu et al., 2021b; Guo et al., 2021). Furthermore, these methods can include biological prior knowledge (e.g., information about known gene association within protein-protein interactions (PPIs) or pathways) (Wei et al., 2017a; Wei et al., 2017b; Wei et al., 2018; Liu et al., 2019b; Wei et al., 2019; Zeng et al., 2019; Cai et al., 2020; Zhai et al., 2020; Zhu et al., 2020). Finally, gene-based outcomes stand out for their better interpretability and crucial biological consequences.

Many statistical and computational approaches for detecting gene-based GGIs have been established. Peng et al.(Peng et al., 2010) introduced the canonical correlation-based U statistic (CCU). They calculated canonical correlation of two genes in both cases and controls. They next used CCU to calculate the difference in correlation, which revealed the presence of GGIs between the two genes. However, this strategy only considered linear correlation in the study. CCU was then expanded to Kernelized CCU (KCCU) (Yuan et al., 2012; Larson et al., 2013), where the kernel discovered a nonlinear relationship. Emily (Emily, 2016) recently introduced AGGrGATOr, a method that combines *p*-values of interaction tests at the marker-level to assess how a pair of genes interacted, which was a strategy that Ma et al. (Ma et al., 2013) previously utilized to discover interactions under quantitative traits. GBIGM is a non-parametric entropy-based approach suggested by Li et al. (Li et al., 2015b).

In this paper, we propose a new approach called gene-based, gene-gene interaction through a maximal neighborhood coefficient (GBMNC), which uses the maximal neighborhood coefficient (MNC) (Cheng et al., 2020) to identify gene-gene interaction of complex diseases at the gene-level in case-control studies. MNC measures a wide variety of dependence with no bias toward relationship types between two random vectors of arbitrary, but not necessarily equal, dimensions; this is superior to Pearson’s correlation, which only consider linear correlations. We introduced a statistic that uses the difference of MIC in cases and controls as an indicator of occurrence of GGIs, bases on the assumption that the joint distribution of two genes should not be significantly different in case and in control samples if there is no interaction between them (i.e. independent) under complex diseases. In simulation studies, our method exhibited an outstanding performance in recognizing the underlying GGIs at the gene level under a variety of conditions. Its application using real data sets showed accurate identification of GGIs.

## 2 Materials and Methods

The statistical procedure for GBMNC is described in depth in this section. We give different parameter settings for simulation studies to evaluate the power to identify GGIs and the ability to control type-I error. Then, we adopted a real-world Rheumatoid Arthritis data set from the WTCCC (Wellcome Trust case Control Consortium) database to evaluate out method’s effectiveness in a real situation.

### 2.1 GBMNC

#### 2.1.1 Preliminaries and Notation

Here, we take genes, a couple of SNPs, as the basic unit. Suppose that we have n random samples:

$$\left(G_{1,i},G_{2,i}\right)\in {ℛ}^{p+q},i=1,2,\ldots ,n$$ (1)

where

$$G_{1,i}=\left(g_{1,i,1},g_{1,i,2},\ldots ,g_{1,i,p}\right),G_{2,i}=\left(g_{2,i,1},g_{2,i,2},\ldots ,g_{2,i,q}\right),\;i=1,2,\ldots ,n$$

and $G_{1}$ and $G_{2}$ represent two genes each with p and q SNPs, independently. In the case-control studies, $y_{i}\in \left\{0,1\right\}$ is a categorical label where 0 is a control subject and one is a case subject. $g_{k,i,j}\in \left\{0,1,2\right\}$ represents the copy number of the minor alleles of SNP j in gene k for sample i .

In this work, to investigate whether there is a statistical interaction between two genes in a qualitative phenotype, we designed a statistic based on the maximal neighborhood coefficient to characterize the GGI intensity. We applied a permutation strategy to estimate the distribution of the statistic. Our approach was based on the intuition that, if there was no interaction between two genes, then, if they were independent of the case set, they should be independent of the control set; if they were dependent on the case set, they should be dependent on the control set as well, and the “strength” of such dependence should be the same for the case and control sets. Pearson’s correlation coefficient measures the degree of dependence between two random variables. However, it can only measure linear dependency and not nonlinear dependency, and it is not very convenient for random variables that take a value in ${ℛ}^{n}$ . Therefore, we proposed to measure dependency between random variables by the maximal neighborhood coefficient (MNC) instead.

#### 2.1.2 Maximal Neighborhood Coefficient

MNC is an association measure that decipher the potential complex associations from neighborhood insight. It assumes that if a relationship exists between two variables, the samples of each variable will appear to have a similar neighborhood tendency to approximate that relationship, and MNC can find those common neighborhood structures by exploring the possible neighborhoods of each variable. By introducing a k -NN granule to reconstruct samples, and a novel neighborhood mutual information (NMI) to measure the certainty information of one variable from another under a fixed $\left(k_{x},k_{y}\right)$ neighborhood combination, MNC enables us to detect more complex associations.

Let $S=\left\{\left(x_{1},y_{1}\right),\ldots ,\left(x_{n},y_{n}\right)\right\}\in {ℛ}^{2}$ be a finite set that is sampled from a joint distribution (X,Y) , and $S_{X}=\left\{x_{1},\ldots ,x_{n}\right\}$ and $S_{Y}=\left\{y_{1},\ldots ,y_{n}\right\}$ represents samples from marginal variables X and Y , respectively. Given a designated neighborhood combination $\left(k_{x},k_{y}\right)$ (a pairwise positive integer), $N_{X}^{k_{x}}\left(x\right)=\left\{x_{j_{1}},\ldots ,x_{j_{k_{x}}}\right\}$ designed as the $k_{x}$ -NN granule of x , where the subscript sequence $j_{1}<j_{2}<\ldots <j_{k_{x}}$ is obtained by $d\left(x,x_{j_{i}}\right)={\left\|x-x_{ji}\right\|}_{2}$ . All samples of $k_{x}$ -NN granules form a cover of $S_{X}$ , that is $\cup_{i=1}^{n}N_{X}^{k_{x}}\left(x_{i}\right)=US_{X}$ . At the same time, there exists a cover for $S_{Y}$ , $\cup_{i=1}^{n}N_{Y}^{k_{y}}\left(y_{i}\right)=S_{Y}$ . The cover of samples S under $\left(k_{x},k_{y}\right)$ is recorded as $C_{k_{x},k_{y}}$ . Let $S{|}_{C_{k_{x},k_{y}}}$ represents the distribution of S on the cover $C_{k_{x},k_{y}}$ , and different neighborhood combinations produce different distributions.

MNC is defined based on the neighborhood characteristic matrix (NM) of a sample set S . Given a finite data set S and a neighborhood combination $\left(k_{x},k_{y}\right)$ , the element of NM of S is:

$$NM{\left(S\right)}_{k_{x},k_{y}}=\frac{NMI\left(S{|}_{C_{k_{x},k_{y}}}\right)}{log\frac{n}{\text{max}\left(k_{x},k_{y}\right)}}$$ (2)

$NMI\left(S{|}_{C_{k_{x},k_{y}}}\right)$ denotes the neighborhood mutual information of distribution $S{|}_{C_{k_{x},k_{y}}}$ . The neighborhood mutual information of ( $x_{i},y_{i}$ ) is defined as follow:

$$NMI_{C_{k_{x},k_{y}}}\left(x_{i},y_{i}\right)=-log\frac{n|N_{X}^{k_{x}}\left(x_{i}\right)\cap N_{Y}^{k_{y}}\left(y_{i}\right)|}{k_{x}k_{y}}$$ (3)

Based on the equation above, the neighborhood mutual information of (X,Y) is defined as:

$$NMI_{C_{k_{x},k_{y}}}\left(X,Y\right)=-\frac{1}{n}\overset{n}{\underset{i=1}{\sum }}log\frac{n|N_{X}^{k_{x}}\left(x_{i}\right)\cap N_{Y}^{k_{y}}\left(y_{i}\right)|}{k_{x}k_{y}}$$ (4)

With the definition of NM(S) in Eq. 2, NMC is defined as:

$$NMC\left(S\right)=\underset{1\leq k_{x}k_{y}\leq NB\left(n\right)}{\max}\left\{NM{\left(S\right)}_{k_{x},k_{y}}\right\}$$ (5)

where NB(n) is the search range, and $1\leq k_{x}k_{y}\leq O\left(n^{\alpha }\right)$ for some 0<α<1 . It also naturally extends to the case of two random vectors with arbitrary, but not necessarily equal, dimensions.

MNC Satisfies the Following Properties

1) Symmertry: MNC(X,Y)=MNC(Y,X) ;
2) Comparability: MNC∈[0,1] , MNC=0 denotes that two variables are statistically independent; MNC=1 implies a strong association between two variables.
3) Generality: MNC captures comprehensive range relationships.
4) Equitability: MNC is robust to noisy relationships. It provides similar scores to the equally noisy relationships of different types.

#### 2.1.3 Illustration of the GBMNC Workflow

Assume there are $n_{1}$ control samples and $n_{2}$ case samples in a case-control study for a pair of genes such that $G_{1}$ has  p SNPs and $G_{2}$ has q SNPs. Let $MNC_{n}\left(G_{1},G_{2}\right)$ be the sample association score between $G_{1}$ and $G_{2}$ . First, we calculate the $MNC_{n_{1}}^{C}\left(G_{1},G_{2}\right)$ for control samples and $MNC_{n_{2}}^{D}\left(G_{1},G_{2}\right)$ for case samples. Second, we design a statistic $\Delta MNC=\;\frac{\left|MNC_{n_{1}}^{C}\left(G_{1},G_{2}\right)-MNC_{n_{2}}^{D}\left(G_{1},G_{2}\right)\right|}{MNC_{n_{2}}^{D}\left(G_{1},G_{2}\right)}$ to measure the difference in MNC between cases and controls. ΔMNC represents how different the two joint distributions $\left(G_{1}^{C},G_{2}^{C}\right)$ and $\left(G_{1}^{D},G_{2}^{D}\right)$ are. The larger the ΔMNC , the higher the probability that $G_{1}$ and $G_{2}$ interact.

To get a *p*-value, we needed to estimate the distribution of $\Delta MNC^{0}$ under the null hypothesis. Here, we used a non-parametric strategy based on permutation: we shuffled the label y randomly m times, calculated ΔMNC using the same procedure above, and used the resulting empirical distribution as an estimate for the distribution of ΔMNC under the null hypothesis. Let the result of these m permutations be $\Delta MNC^{1},\ldots ,\Delta MNC^{m}$ , then an estimated *p*-value for the null hypothesis is

$$p=\frac{\left|\left\{i:\Delta MNC^{i}\geq \Delta MNC^{0}\right\}\right|}{m}$$ (6)

**FIGURE 1:**
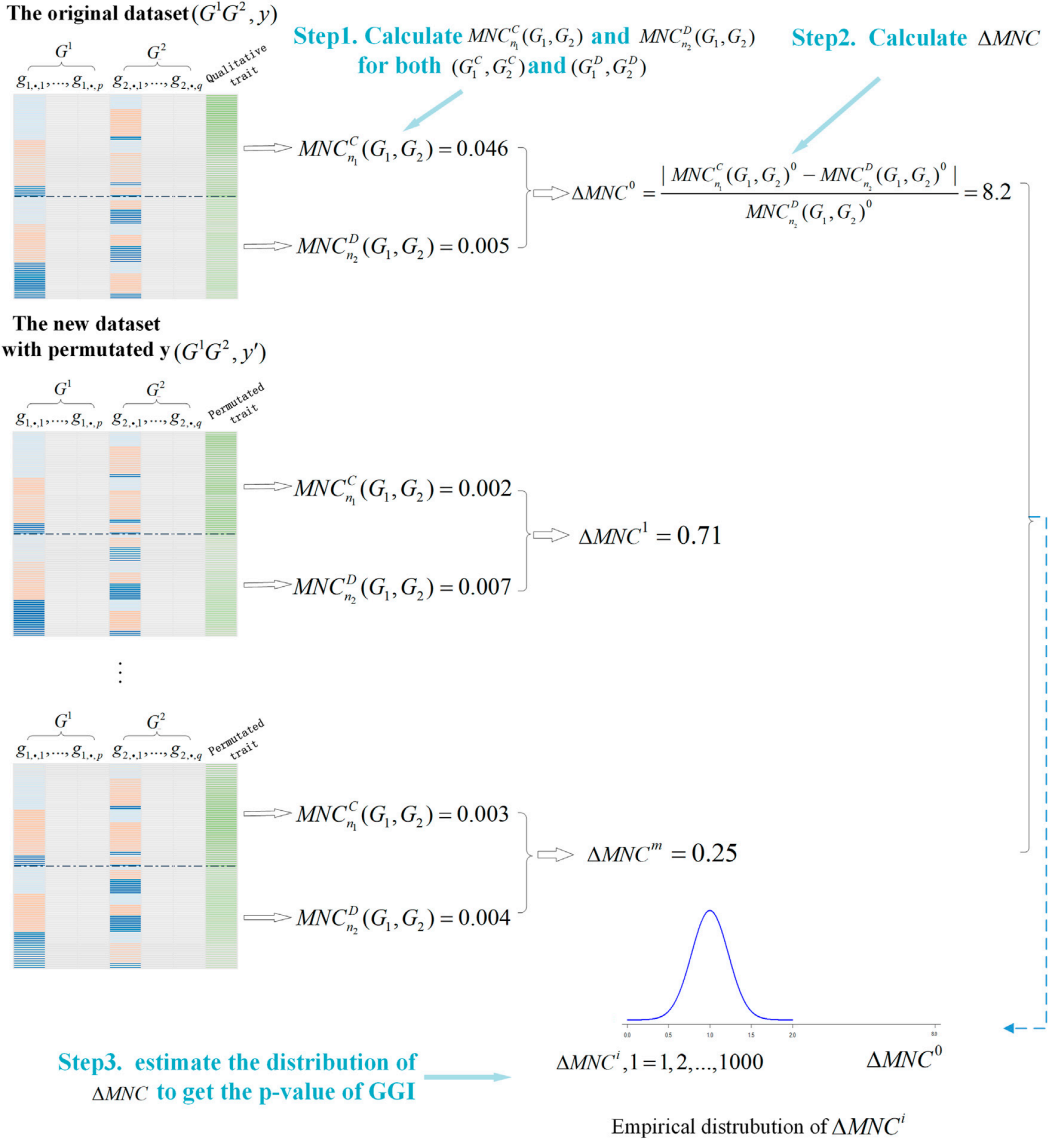
Illustration of the Gene-Based gene-gene interaction through a Maximal Neighborhood Coefficient (GBMNC) workflow for detection of gene-based, gene-gene interaction.

We summarized the process of GBMNC in the algorithm below (Algorithm 1) and presented the overall workflow (Figure 1).

Algorithm 1 **GBMNC**

**Data**: Genotype $G_{1},G_{2}$ , Phenotype y , permutation times m

**Result**: significant p-value for interaction between $G_{1},G_{2}$

1  Calculate $MNC_{n_{1}}^{C}\left(G_{1},G_{2}\right)$ and $MNC_{n_{2}}^{D}\left(G_{1},G_{2}\right)$ for both $\left(G_{1}^{C},G_{2}^{C}\right)$ and $\left(G_{1}^{D},G_{2}^{D}\right)$ by Eq. 5;
2  Calculate the difference $\Delta MNC^{0}$ between $MNC_{n_{1}}^{C}\left(G_{1},G_{2}\right)$ and $MNC_{n_{2}}^{D}\left(G_{1},G_{2}\right)$ ;
 3 **for** i=1 to m **do**
  4 Randomly permute label y , and generate the new data set;
  5 Repeat Steps 1 and 2;
  6 **end**
  7 Estimated *p*-value of $\Delta MNC^{0}$ is the number of $\Delta MNC^{i}$ , i=1,…,m , which are larger than $\Delta MNC^{0}$ , divided by m .

### 2.2 Simulation Study

To assess the performance of GBMNC to control type I error and the power to detect GGIs, we compared GBMNC with KCCA (Larson et al., 2013), GBIGM (Li et al., 2015b), and AGGrEGATOr (Emily, 2016).

#### 2.2.1 Simulation With GAMETES

The goal of this simulation study was to evaluate the performance of the GBMNC procedure to detect gene-gene interaction. We set all simulated datasets to have 50 SNPs. Among them, two SNPs were functional, and the remaining 48 SNPs were non-functional. The 50 SNPs formed five genes, and each had 10 SNPs. The two functional SNPs were put into the first and second genes. We chose the publicly available tool GAMETES (Urbanowicz et al., 2012) to generate the simulated genotype data. This tool was designed to generate pure and strict epistasis models. Pure and strict epistasis models are the most difficult disease-related patterns to identify. Such associations can only be observed if all n-loci are included in the disease model. This requirement makes these types of models an attractive gold standard for simulation studies of complex multi-locus effects.

*Evaluation of Type-I error*: The type-I error indicates the ability of a method to reject the null hypothesis when it is true (i.e., the false positive rate). We used GAMETES to generate the custom disease model (Table 1) with one causal SNP pair. γ characterizes the baseline odds (i.e., the odds conditional on genotype pair AABB ). We ran the simulation 100 times with each sample size n∈{1k,2k,3k,4k,5k} and γ=1 . The significance level α was set to be 0.05.

**TABLE 1:** Table of odds for the no effect model without interaction between a pair of SNPs.

	AA	Aa	Aa
**BB**	γ	γ	γ
**Bb**	γ	γ	γ
**bb**	γ	γ	γ

*Evaluation of power of the test*: The power of a test indicates the probability that the method rejects the null hypothesis correctly when the alternative hypothesis is true. In this simulation study, we generated 100 data sets for each parameter settings. The power under each parameter setting was expressed by the frequency, and the null hypothesis of the data set was rejected correctly at the significance level of α=0.05 .

1) To assess the impact of heritability h , which measured the intensity of correlation between genotype and phenotype, we chose h∈{0.01,0.025,0.05,0.1,0.2} and two different minor allele frequencies MAF ∈{0.2,0.4} with population prevalence set to 0.2 and sample size set at 4,000. Under each parameter combination, five models were generated so that we had a total of 100 models that followed Hardy-Weinberg proportions. For a specified genetic constrain combination, the 10 models were sorted roughly by the ascending customized odds ratio (COR) using GAMETES and labeled M1 to M5. COR is a metric of detectability that was calculated directly from the genetic model. The higher it is, the easier it is to detect GGIs. GAMETES generated the penetrance tables for these 100 models in the absence of the main effect. One hundred replicated data sets were generated from each model with balanced cases and controls, which resulted in 5,000 data sets in total in this scenario.
2) To evaluate the influence of sample size, we set heritability to be 0.025, MAF ∈ {0.2,0.4} and prevalence to be 0.2 with a sample size of 10,000. Then, 100 data sets were generated by random sampling from this large dataset for each of the sample sizes n∈{1k,2k,3k,4k,5k} . In this scenario, we had 1,000 datasets in total.

For GBMNC, KCCU, AGGrEGATOr, and GBIGM, if the number of data sets with a significance level less than α  is $m_{1}$ , then the power can be calculated by the following formula:

$$power=\;\frac{m_{1}}{100}$$ (7)

GBIGM and AGGrEGATOr methods are nonparametric methods, so no parameters need to be specific. We only set the ratio of the trimmed jackknife to 0.05 ( ω=0.05 ) for KCCU.

## 2.3 Experiments Using Rheumatoid Arthritis Data

To evaluate GBMNC’s ability to process real GGIs in a qualitative data set, we analyzed the susceptibility of a series of pairs of genes in Rheumatoid Arthritis (RA). RA is a chronic autoimmune disease that causes pannus development and cartilage and bone loss in synovial joints. It leads to progressive bone deterioration and interferes with bone repair. In this work, we used the WTCCC (2007) data set, which includes genotype data from the British population obtained by the Affymetrix GeneGhip 500 k. Our dataset was pre-processed in the following ways:

1) We used pathway hsa05323 from the KEGG pathway database to validate the GGIs in the RA. The WTCCC data set’s genotyping coordinates can be found in UCSC hg18/NCBI Build36. This pathway contained 90genes. Many of the genes belonged to the protein combinations MHCII and V-ATPase. Because numerous GGIs happened on their own, we only chose representative genes from each protein combination and then remove the others. Finally, 48genes remained, resulting in a total of $C_{48}^{2}=1128$ pairs of genes to be analyzed.
2) We collected the detailed gene information from the NCBI Build36 annotation file, and for each gene, we inserted a 10 kb buffer region both downstream and upstream of the originally defined gene location. For each gene, all SNPs within the area were chosen.
3) According to the quality control of GWAS, samples that included gender that did not match the chromosome X heterozygote rates were removed. SNPs were also removed if any of the following requirements were met: the missing rate in the sample was ≥10% , MAF was ≤0.05 , or the frequency of control violated Hardy-Weinberg equilibrium ( p<0.0001 ). Finally, 385 SNPs remained in 4,966 samples, which included 2,993 control subjects and 1973 case subjects.

## 3 Results and Discussion

The experimental environment for all the following results was a workstation with an Intel Xeon CPU E5-2,620 v2 at 2.10GHz, 96 GB of DDR3, and python3.6.

### 3.1 Simulation Study

#### 3.1.1 Evaluation of Type-I Error

For type-I error, we varied the sample size from 1,000 to 5,000. Except for GBIGM with n=1,000 , all methods tested had a type-I error comparable to a significance level α=0.05 (Table 2), which implied that these methods controlled for type-I error for various sample sizes quite well.

**TABLE 2:** Type-I error for KCCU, GBIGM, AGGrEGATOr, and GBMNC when varying the sample size from 1,000 to 5,000.

Methods	Sample size				
	1,000	2,000	3 *,*000	4,000	5,000
**KCCU**	0.02	0.02	0.01	0.05	0.07
**GBIGM**	0.13	0.06	0.07	0.07	0.07
**AGGrEGATOr**	0.05	0.06	0.07	0.04	0.02
**GBMNC**	0.02	0.05	0.07	0.05	0.05

#### 3.1.2 Evaluation of the Power of GBMNC

*Impact of heritability*: To evaluate the statistical power of our GBMNC and the other three methods, we used 10 heritability-MAF combinations, with a population prevalence of 0.2, a sample size of 4,000, and heritability that varied from 0.01 to 0.2 (Table 3). The bold in Table 3 shows the best-performed method in each model under a given heritability-MAF combination. Notice that a larger value indicates better performance. On average, GBMNC was the best performing algorithm in this comparison. It largely outperformed the other methods, but not for all the data sets; it was inferior to AGGrEGATOr for some data sets. However, its performance was remarkably consistent, and it was the top performer for most data sets. AGGrEGATOr achieved the same performance when MAF was 0.2 and heritability was >0.05.

**TABLE 3:** The statistical power of simulation studies for GBMNC, AGGrEGATOr, KCCU and GBIGM under 10 heritability-MAF combinations, with h∈{0.01,0.025,0.05,0.1,0.2} and MAF ∈{0.2,0.4} . Each heritability-MAF combination has five models. Bold font indicates the method that performed best under each model.

MAF	Heritability	Model	M1	M2	M3	M4	M5
		**Method**					
0.2	0.01	GBMNC	0.13	0.40	0.68	0.72	0.89
		AGGrEGATOr	0.12	0.12	0.89	0.89	1
		KCCU	0.15	0.09	0.29	0.43	0.62
		GBIGM	0.09	0.11	0.13	0.11	0.08
	0.025	GBMNC	0.95	0.75	1	0.96	1
		AGGrEGATOr	1	0.27	1	0.37	1
		KCCU	0.58	0.09	0.74	0.24	0.8
		GBIGM	0.08	0.07	0.11	0.13	0.2
	0.05	GBMNC	0.68	0.83	0.94	1	1
		AGGrEGATOr	0.09	0.59	0.89	1	1
		KCCU	0.13	0.57	0.65	0.84	0.85
		GBIGM	0.18	0.08	0.22	0.17	0.19
	0.1	GBMNC	1	1	1	1	1
		AGGrEGATOr	1	1	1	1	1
		KCCU	0.81	0.93	0.9	0.86	0.91
		GBIGM	0.15	0.14	0.23	0.16	0.16
	0.2	GBMNC	1	1	1	1	1
		AGGrEGATOr	1	1	1	1	1
		KCCU	0.89	0.97	0.94	0.89	0.97
		GBIGM	0.19	0.31	0.18	0.22	0.21
0.4	0.01	GBMNC	0.75	0.66	0.82	0.90	0.96
		AGGrEGATOr	0.71	0.09	0.1	0.94	0.96
		KCCU	0.34	0.05	0.08	0.77	0.29
		GBIGM	0.09	0.08	0.1	0.11	0.07
	0.025	GBMNC	1	0.73	0.85	0.93	0.80
		AGGrEGATOr	0.99	0.56	0.12	0.91	0.26
		KCCU	0.58	0.24	0.08	0.24	0.11
		GBIGM	0.15	0.12	0.14	0.11	0.09
	0.05	GBMNC	1	1	1	0.68	0.86
		AGGrEGATOr	1	0.97	0.91	0.35	0.42
		KCCU	0.86	0.9	0.95	0.41	0.37
		GBIGM	0.11	0.12	0.09	0.08	0.10
	0.1	GBMNC	1	1	1	0.63	1
		AGGrEGATOr	0.98	1	0.96	0.27	1
		KCCU	0.62	1	0.95	0.41	1
		GBIGM	0.12	0.19	0.18	0.26	0.20
	0.2	GBMNC	1	1	1	1	1
		AGGrEGATOr	0.93	1	0.99	1	0.80
		KCCU	0.28	1	0.83	1	0.76
		GBIGM	0.19	0.25	0.31	0.13	0.26

The power of all the methods was significantly affected by heritability (i.e., the effect size of interaction) (Table 4). A larger heritability led to better performance for all methods under a specific MAF. When heritability varied from 0.01 to 0.025, GBMNC almost doubled its power for a given sample size of 4,000 with MAF =0.2 . Other methods also show a steady upward trend (Table 4). The power also depended on the MAF of the interacting SNPs (e.g., for the cases of h=0.01 , the power of GBMNC under model M1-M5 ranged between 0.13–0.89 for MAF =0.2 , but it ranged between 0.66–0.96 for MAF =0.4 (Table 3)). The average power was 0.564 for MAF =0.2 , which was much lower than 0.818 for MAF =0.4 (Table 4).

**TABLE 4:** Average power for GBMNC, AGGrEGATOr, KCCU, and GBIGM under 10 heritability-MAF combinations, with heritability ∈{0.01,0.025,0.05,0.1,0.2} and MAF. ∈{0.2,0.4}

MAF	Method	GBMNC	AGGrE-GATOr	KCCU	GBIGM
	Heritability				
0.2	0.01	0.564	0.604	0.316	0.104
	0.025	0.932	0.728	0.490	0.118
	0.05	0.890	0.714	0.608	0.168
	0.1	1	1	0.882	0.168
	0.2	1	1	0.932	0.222
0.4	0.01	0.818	0.560	0.306	0.090
	0.025	0.862	0.568	0.250	0.122
	0.05	0.908	0.730	0.698	0.100
	0.1	0.926	0.842	0.796	0.190
	0.2	1	0.944	0.774	0.228

**FIGURE 2:**
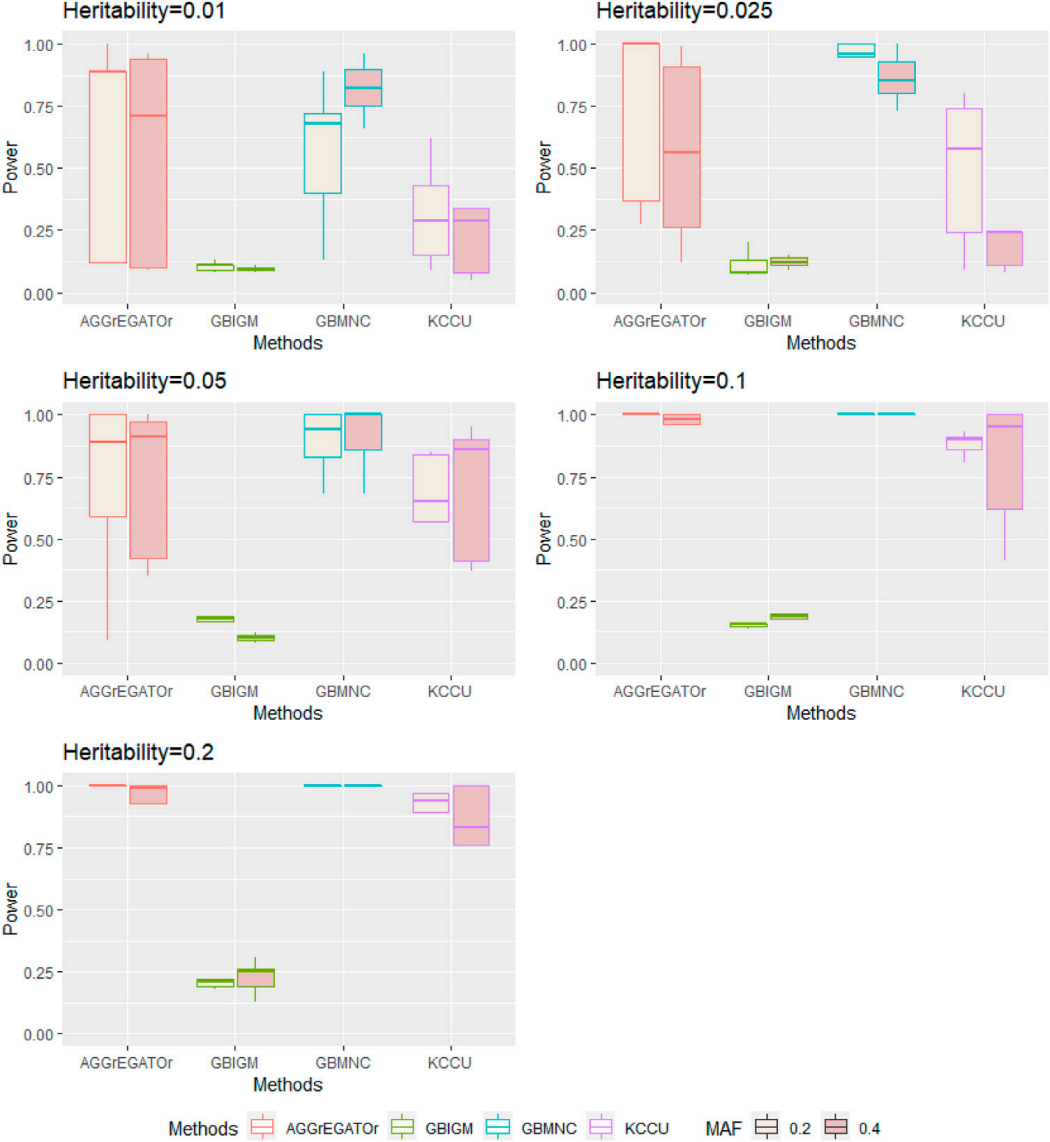
Illustration of the distribution of power of each method in each heritability-MAF combination with h∈{0.01,0.025,0.05,0.1,0.2} and MAF ∈{0.2,0.4} .

It is worth noting that under the same combination of habitability and MAF, GBMNC was more stable under models with different COR compared with AGGrEGATOr (Figure 2). KCCU detected the interaction of some simulated disease models in our study, and it had a similar performance pattern with AGGrEGATOr. However, AGGrEGATOr was much more powerful in most of the simulated scenarios. GBIGM had little power to detecting pure gene-gene interaction,. This result replicated Emily's (Emily, 2016) result of the simulation.

*Impact of sample size*: The sample size of the data set had a considerable effect on power. Let the sample size be n∈{1k,2k,3k,4k,5k} , h=0.025 , and MAF ∈{0.2,0.4} (Table 5). As the sample size increased, the power of all methods increased almost monotonically under different MAF settings. With all methods, a larger sample size corresponded to better performance.

**TABLE 5:** The statistical power of simulation studies for GBMNC, AGGrEGATOr, KCCU, and GBIGM under models with h=0.025 , MAF ∈{0.2,0.4} , and sample sizes that varied from 1k to 5k .

MAF	Method	GBMNC	AGGrEGATOr	KCCU	GBIGM
	Sample size				
0.2	1,000	0.67	0.15	0.11	0.2
	2000	0.83	0.18	0.38	0.16
	3,000	1	0.20	0.55	0.23
	4,000	1	0.31	0.76	0.21
	5,000	1	0.29	0.87	0.12
0.4	1,000	0.68	0.16	0.13	0
	2000	0.97	0.20	0.11	0.04
	3,000	1	0.35	0.2	0.11
	4,000	1	0.54	0.37	0.11
	5,000	1	0.65	0.58	0.05

In conclusion, in simulated studies, our results showed that GBMNC detected gene-gene interaction effectively, in which a pair of SNPs was a causal factor by the purely and strictly epistasis model without main effect, which can only be observed if all 2-loci are included in the disease model. Compared with other methods, GBMNC identified a broad range of epistatic signals accurately.

## 3.2 Experiments Using Rheumatoid Arthritis Data

RA is a chronic autoimmune disease where HLA genes, TNF family, and TRAF1 are important genetic risk factors in the development. Each unique gene pair of the hsa05323 pathway was evaluated in the RA study, which resulted in $C_{48}^{2}=1128$ total pairs for 48 genes. With a significance level α=0.01 and multiple testing adjustment, for KCCU and GIGBM, we obtained 159 and 134 significant GGIs, respectively. Among them, 30 and 65 had *p*-values equal to 0; hence we were unable to rank them in the order of significance. AGGrGETOr did not show any significant results. Following Emily (Emily, 2016), and after removing the multiple testing correction, AGGrGETOr exhibited 17 significant GGIs, which we ranked by their *p*-values. We chose the top 10 gene pairs obtained by GBMNC and by AGGrGETOr to analyze, which comprised approximately 1% of the total interactions (Table 6).

**TABLE 6:** The calculated *p*-value for the 20 gene pairs using GBMNC and AGGrEGATOr. *p*-values in bold font indicate that they are significant. The “Chr” column indicates the chromosome number of the human genome where the gene is located.

Gene1	Chr	Gene2	Chr	*p*-value	
				GBMNC	AGGrEGATOr
TGF- β 2	1	CXCL8	4	0.0	1
CTLA4	2	GM-CSF	5	0.0	0.327
CD80	3	HLA-classII	6	0.0	0.37
GM-CSF	5	TRAP	19	0.0	0.01
TLR-4	9	FLT-1	13	0.0	0.069
IL-17	6	TNFSF13B	13	0.0	0.185
CXCL6	4	ICAM1	19	0.0	1
CD28	2	CXCL6	4	0.0	0.512
CTLA4	2	CXCL6	4	0.0	0.849
MMP-3	11	FLT-1	13	0.0	0.089
CD80	3	April	17	0.99	0.0007
CTSK	1	TNFSF13B	13	0.615	0.0008
JUN	1	IL-6	7	0.445	0.0019
CD80	3	CTSL	25	0.0	0.002
CXCL6	4	FLT-1	13	0.297	0.0021
CTLA4	2	FOS	37	0.727	0.0022
FLT-1	13	LFA-1	39	0.815	0.0033
CCL3	17	TRAP	19	0.564	0.0034
IL-18	11	TGF- β 3	14	0.693	0.004
IL-1	2	CXCL12	10	0.081	0.004

We found that some of our findings were supported by prior research (Xiao et al., 2008; Klocke et al., 2016; Cen et al., 2019). For instance, our method detected a significant interaction between IL17 and TNFSF13B. Studies (Xiao et al., 2008) show that both B cells and T cells formed aggregates in the synovium of inflamed joints and mediated the pathogenesis of RA, and B-cell-activating factor (BAFF, also named TNFSF13B, BLys) played a vital role in B-cell survival and maturation. After activation and expansion, CD4^+^ T cells developed into different T helper cell subsets with different cytokine profiles and distinct effector functions. In addition to Th1 and Th2 cells, Th17 cells were a third T helper cell and produce IL-17. Th17 cells can recruit and activate inflammatory cells and they have been recognized as a primary cause of bone destruction and inflammation in autoimmune diseases. BAFF promoted Th17 cell proliferation and expansion preferentially (Lai Kwan Lam et al., 2008). IL-17 was a key cytokine for BAFF-mediated proinflammatory effects during collagen-induced arthritis pathogenesis. Only one pair of potential interactions between CD80 and CTSL was captured by both methods within the top 10 GGIs. However, there is not yet direct evidence to show the interaction between CD80 and CTSL.

## 4 Conclusion

The study of detecting GGIs is of great importance in understanding the pathogenesis of complex human diseases. In this paper, we proposed a gene-based GGI detection method called GBMNC based on a maximal neighborhood coefficient and a permutation strategy for case-control studies in GWAS. The method not only benefited from the ability of a maximal neighborhood coefficient, which considered the neighborhood structure of each sample and captured a wide range of associations, but also from the robustness of our permutation-based hypothesis testing scheme.

We designed a statistic to capture the different intensities of interaction between two genes in both cases and controls, then transformed the problem of GGI detection into a form of hypothesis testing; our null hypothesis was there was no significant difference in the relationship between the two genes in the disease data and the control data. This hypothesis did not limit the form of interaction between genes, and it enhanced the method’s ability to detect different types of interactions. We demonstrated the effectiveness of our method through a simulation study and retrospective analysis of rheumatoid arthritis. Under a large range of settings, GBMNC outperformed previous methods in the power to detect GGIs. The statistical power of our method increased monotonically with the increase in the heritability and the MAF. The method was also stable to sample size based on a test of false positive rates. MNC did not restrict the dimension of two random vectors. Therefore, it is possible to generalize the method for marker-based detection of gene pairs that are identified as interactive. Investigating the mechanism of gene-based interaction at the marker level might point the way for further research. In summary, GBMNC is a helpful addition to the current toolbox of statistical models to elucidate GGIs in case-control studies.

## Data Availability

Publicly available datasets were analyzed in this study. This data can be found here: https://www.wtccc.org.uk/info/access_to_data_samples.html.
